# Multiple genome-wide analyses of smoking behavior in the Framingham Heart Study

**DOI:** 10.1186/1471-2156-4-S1-S102

**Published:** 2003-12-31

**Authors:** Ellen L Goode, Michael D Badzioch, Helen Kim, France Gagnon, Laura S Rozek, Karen L Edwards, Gail P Jarvik

**Affiliations:** 1 Cancer Prevention Research Program, Fred Hutchinson Cancer Research Center, Seattle, Washington, USA; 2 Division of Medical Genetics, University of Washington Medical Center, Seattle, Washington, USA; 3 Department of Epidemiology, University of Washington, Seattle, Washington, USA; 4 Department of Epidemiology and Community Medicine, University of Ottawa, Ottawa, Ontario, Canada; 5 Department of Epidemiology, University of Michigan, Ann Arbor, Michigan, USA; 6 Institute of Public Health Genetics, University of Washington, Seattle, Washington, USA

## Abstract

**Background:**

Cigarette smoking behavior may have a genetic basis. We assessed evidence for quantitative trait loci (QTLs) affecting the maximum number of cigarettes smoked per day, a trait meant to quantify this behavior, using data collected over 40 years as part of the Framingham Heart Study's original and offspring cohorts.

**Results:**

Heritability was estimated to be approximately 21% using variance components (VC) methods (SOLAR), while oligogenic linkage and segregation analysis based on Bayesian Markov chain Monte Carlo (MCMC) methods (LOKI) estimated a mean of two large QTLs contributing approximately 28% and 20%, respectively, to the trait's variance. Genome-wide parametric (FASTLINK) and VC linkage analyses (SOLAR) revealed several LOD scores greater than 1.0, with peak LOD scores using both methods on chromosomes 2, 17, and 20; multi-point MCMC methods followed up on these chromosomes. The most robust linkage results were for a QTL between 65 and 84 cM on chromosome 20 with signals from multiple sex- and age-adjusted analyses including two-point LOD scores of 1.30 (parametric) and 1.07 (heritability = 0.17, VC) at 70.51 cM, a multi-point LOD score of 1.50 (heritability = 0.20, VC) at 84 cM, and an intensity ratio of 12.0 (MCMC) at 65 cM.

**Conclusion:**

Familial aggregation of the maximum number of cigarettes smoked per day was consistent with a genetic component to this behavior, and oligogenic segregation analyses using MCMC suggested two important QTLs. Linkage signals on chromosome 20 between 65 and 84 cM were seen using multiple analytical methods. No linkage result, however, met genome-wide statistical significance criteria, and the true relationship between these regions and smoking behavior remains unclear.

## Background

Many behaviors, such as smoking, offer a variety of possible phenotypes that may have differing genetic components. Genomic scans for current smoking status [1,2], pack-years smoked [2], and nicotine dependence [3] and association studies for a variety of smoking-related behaviors have been conducted. Longitudinal data offer additional phenotypes, including the maximum number of cigarettes smoked per day over several years, a trait which may be more genetic than current smoking habits. We assessed evidence for the existence and localization of quantitative trait loci (QTLs) for "maximum number of cigarettes smoked per day" using data collected over 40 years in the Framingham Heart Study and the Framingham Offspring Study.

## Methods

### Study subjects and data collection

Data from the Framingham Heart Study and the Framingham Offspring Study were analyzed as part of Genetic Analysis Workshop 13 (GAW13) and are described elsewhere [4,5].

### Phenotypes

Self-reported number of cigarettes smoked per day was available from at least one exam for 2883 participants. The quantitative trait maximum number of cigarettes per day represented the largest number of cigarettes smoked per day reported by each participant at any point throughout the study; this value was equal to zero for individuals who reported smoking no cigarettes at each exam. Skewness and kurtosis for maximum number of cigarettes per day were 1.0 and 3.7, respectively (excluding non-smokers these were 0.8 and 4.3, respectively). A variety of transformations were used on the trait; however, because none markedly reduced skewness or kurtosis, analyses were performed on untransformed values. Covariates for some analyses included sex and the age and year at which the maximum number of cigarettes were smoked. These age and year variables were the first age and year at which the person reported smoking that quantity (including zero for non-smokers) if it was reported more than once.

### Familial correlations, heritability estimation, and segregation analyses

Intraclass correlation coefficients among pairs of relatives were calculated using FCOR, a component of S.A.G.E. 4.2 [6] with pedigrees weighted equally. Heritability estimates for maximum number of cigarettes per day were obtained using the variance-components (VC) approach implemented in the SOLAR package 1.7.3 [7], which partitions the total phenotypic variance into additive genetic variance attributable to the QTL, residual polygenic additive genetic variance, and variance due to random environmental effects. Oligogenic joint linkage and segregation analysis was performed using Bayesian Markov chain Monte Carlo (MCMC) methods implemented in LOKI 2.2 [8], with sex and age as covariates.

### Linkage analyses

Genome-wide two-point parametric LOD score analyses for maximum number of cigarettes per day were performed with FASTLINK [9-11] using gene frequency and genotype-specific phenotypic mean estimates provided by analyses in PAP 4.0 [12] and S.A.G.E. 4.0 [6], which assumed the existence of a major gene. Parametric analyses were performed on unadjusted values and on residuals from a linear regression of maximum number of cigarettes per day on sex and age.

Genome-wide two-point VC linkage analysis was performed using SOLAR 1.7.3 [7]. Under the null hypothesis of no linkage, the QTL variance was fixed at zero and was tested against a polygenic model in which the same parameter was estimated from the data using maximum likelihood methods. Adjusted VC analyses considered sex and age as covariates. Adjusted multi-point analysis was performed on chromosomes with sex- and age-adjusted two-point LOD scores > 1.0.

In order to assess possible cohort effects, additional two-point parametric LOD and VC analyses considered year of smoking maximum number of cigarettes as a covariate.

Chromosomes yielding LOD scores > 1.0 in both parametric and VC sex- and age-adjusted analyses were followed up with multi-point oligogenic joint linkage and segregation analyses using Bayesian MCMC methods implemented in LOKI 2.2 [8] with sex and age as covariates. The effect of non-normality has not been reported in the context of LOKI's MCMC approach; therefore, the trait values for non-smokers were treated as unknown so that the data were less skewed. The initial estimates (the "priors") for the number of QTLs and the tau beta (i.e., variance in the genotypic effects) were set at 2 and 20, respectively, based on oligogenic segregation analyses, and a limit on the residual variance was set at 75 to improve mixing (i.e., allowing the sampler to visit various parts of the sample space for the parameter estimates). We defined a "large QTL" as a locus with an individual contribution of at least 5% of the total variance of the trait. Since the Bayesian analysis method used does not provide traditional LOD scores or *p*-values, the results were used to provide a count of the number of times a particular genomic position was accepted as the position of a QTL during an update of the model (a "hit"). Using the intensity ratio (IR), these estimated numbers of hits were then compared with the number that would be expected by chance, given the specified prior distributions. In map interval *i*, the IR_*i *_was calculated as follow: IR_*i *_= *h*_*i*_*/e*_*i*_, where *h*_*i *_was the observed number of hits in map interval *i *and *e*_*i*_, the expected number of hits in map interval *i*. The expected number of hits *e*_*i *_being *(E(n)/L) *× *b*_*i *_× *I*. *E(n) *was the expected number of QTLs in an iteration in the analysis, obtained from the prior distribution, *L *was the total map length, *b*_*i *_was the bin width, and *I *was the number of iterations. IRs were computed using a 2-cM bin and a total map length of 3000 cM.

## Results

Of 330 pedigrees (4692 individuals), smoking history was available for 2883 individuals, including 1743 (37%) ever-smokers (653 genotyped) and 1140 (24%) never-smokers (402 genotyped). Among smokers, on average, the amount of smoking when individuals smoked their most was 24.2 cigarettes per day (range, 1–95) occurring at an average age of 41.7 years (range, 15–82 years) in 1967 (range 1948–1991).

### Familial correlations, heritability estimation, and segregation analyses

Maximum number of cigarettes smoked per day was correlated in sibling pairs (r^2 ^= 0.18 ± 0.03, 2796 pairs); the correlation estimate did not vary after adjustment for age and sex (r^2 ^= 0.18 ± 0.03) or age, sex, and year (r^2 ^= 0.16 ± 0.03). Unadjusted correlation estimates were lower for parent-child pairs (r^2 ^= 0.09 ± 0.03, 3037 pairs) and spouse pairs (r^2 ^= 0.13 ± 0.04, 486 pairs). Heritability of this trait was estimated to be 0.21 (SE = 0.03) using the VC approach (*p *< 0.001), and did not vary with adjustments for sex, age, or year (range, 0.21–0.23). Allowing for a t-distribution did not change VC modeling results (heritability range, 0.21–0.23). Using MCMC oligogenic segregation analysis, the components of variance for maximum number of cigarettes smoked per day were as follows: residual variance 41%, age 3%, sex 5%, and total genetic variance 51%. Two large QTLs were estimated for the trait, with individual contributions of approximately 28% and 20% of the total variance, respectively, which explained approximately 55% and 39% of the genetic variance. MCMC analysis estimated the largest QTL to be overdominant.

### Linkage analyses

**Figure 1:**
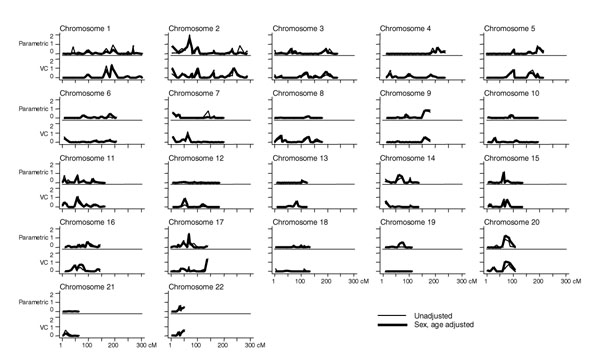
**Two-point parametric and variance components (VC) LOD scores for maximum number of cigarettes per day. **Parametric LOD scores are maximized over θ; cM = sex-averaged distance from p-term.

Genome-wide two-point parametric LOD scores for maximum number of cigarettes smoked per day are shown in Figure 1 (upper graphs) for both the unadjusted trait and for sex- and age-adjusted residuals (age and sex were significant covariates in linear regression, *p *< 0.001). There were two LOD scores greater than 1.0 in unadjusted analyses; one on chromosome 2 at 70.32 cM (LOD = 1.98) and the other on chromosome 17 at 66.85 cM (LOD = 1.52) (Table 1). Adjustment for sex and age did not appreciably change these results. However, additional peaks in sex- and age-adjusted analyses were seen on chromosome 15 at 65.52 cM (LOD = 1.09) and on chromosome 20 (consecutive markers at 70.51 and 84.62 cM, LODs = 1.30 and 1.02, respectively). With the additional inclusion of year at smoking maximum number of cigarettes, chromosome 20 LOD score peaks remained, however chromosome 2 and 17 peaks seen in unadjusted LOD score analysis were reduced (Table 1).

Results of VC genome-wide linkage analyses are graphed in Figure 1 (lower graphs) as well. Five two-point LOD scores greater than 1.0 were observed, including three markers within 26 cM on chromosome 1: at 167.15 cM (LOD = 1.28), at 187.86 cM (LOD = 1.46), and at 193.02 cM (LOD = 1.13). Other elevated unadjusted LOD scores were seen on chromosome 7 at 62.68 cM (LOD = 1.10), and chromosome 17 at 138.03 cM (LOD = 1.12). Sex and age were found to be significant covariates in the VC model (*p *< 0.001). Adjustment for sex and age reduced the significance of the signal on chromosome 1 (Table 1), though a peak LOD score of 1.34 at 187.86 cM remained. Additional LOD scores greater than 1.0 in sex- and age-adjusted analyses were seen on chromosomes 2, 11, 17, and 20 (Table 1). Two consecutive chromosome 20 markers at 70.51 and 84.62 cM that had elevated LOD scores in adjusted parametric analyses also had elevated LOD scores in adjusted VC analyses (LOD = 1.07 and 0.95, respectively). Additional consideration of year at smoking maximum number of cigarettes further increased these chromosome 20 peaks (LOD = 1.39 and 0.98, respectively), though chromosome 1 peaks were further reduced (Table 1). Multi-point sex- and age-adjusted VC analyses yielded peak LOD scores of 1.71 (241 cM on chromosome 2) and 1.50 (84 cM on chromosome 20), but no LOD scores greater than 1.0 were observed on chromosomes 1, 11, or 17.

**Figure 2:**
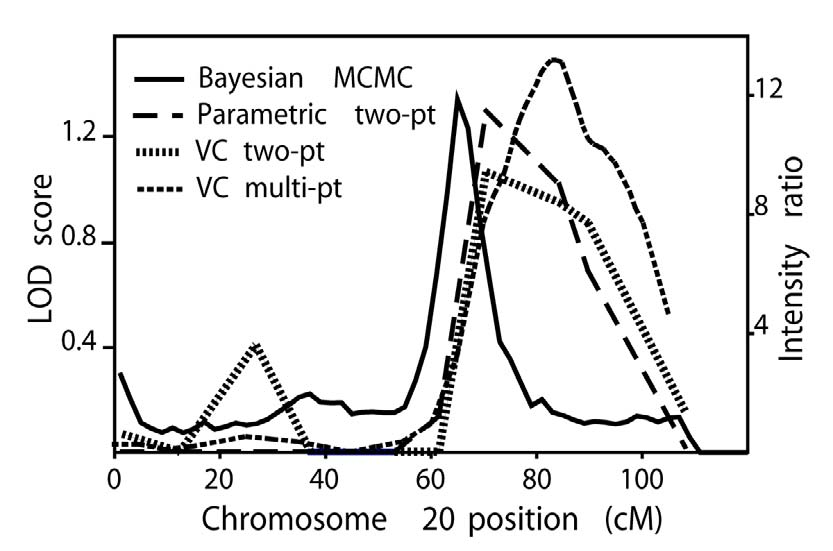
Chromosome 20 results from sex- and age-adjusted analyses of maximum number of cigarettes per day.

As follow-up on the common signals obtained with parametric and VC methods, we analyzed chromosomes 2, 17, and 20 using MCMC methods. Using sex- and age-adjusted joint linkage and segregation analysis, evidence of linkage for maximum number of cigarettes smoked per day was not seen on chromosome 17. However, signals were identified on chromosomes 2 and 20, with IRs of less than 2.0 at approximately 149 cM on chromosome 2, and approximately 12.0 at 65 cM on chromosome 20. Figure 2 displays MCMC intensity ratios with VC and parametric LOD scores for chromosome 20 where multiple analyses revealed peaks between 65 and 84 cM.

## Conclusions

In summary, a quantitative trait representing the maximum (over several years) number of cigarettes per day in the Framingham Heart Study's original and offspring cohorts was assessed in segregation and linkage analyses. Correlations between sibling pairs were consistent with the existence of a genetic component, as were variance components estimates of heritability (0.21–0.23); in addition, MCMC oligogenic segregation analysis estimated that 48% of the total phenotypic variance could be attributable to approximately two large QTLs.

Genome-wide linkage analyses using parametric and VC methods, both unadjusted and adjusted for sex and age, revealed several regions with LOD scores greater than 1.0. Chromosomes 2, 17, and 20 harbored peak LOD scores using both methods (though only in similar positions on chromosome 20). Follow-up of these chromosomes using multi-point MCMC analysis were consistent with the existence of QTLs on chromosomes 2 and 20. The most robust linkage result in our analyses was between 65 and 84 cM on chromosome 20 in the 20q13.1 region; results from sex- and age-adjusted analyses included peak two-point LOD scores of 1.30 (θ = 0.2, parametric) and 1.07 (heritability = 0.17, VC) at 70.51 cM (marker GATA47F05), a peak multi-point LOD score of 1.50 (heritability = 0.20, VC) at 84 cM, and an IR of 12.0 (MCMC) at approximately 65 cM. The trait distribution's non-normality may have affected accuracy of QTL detection, however, and no linkage result in this analysis was statistically significant.

We chose to examine the genetics underlying a propensity to smoke large quantities of cigarettes at any age, a narrowly defined "extreme" quantitative phenotype that we hoped may have provided improved power [1-3]. Nonetheless, difficulties in assigning phenotypes remained; for example, the inclusion of non-smokers as having trait equal to zero may not have improved power to detect linkage if non-smoking individuals harbored an untriggered propensity for addiction. Because smoking patterns changed dramatically over the last half-century, we also hypothesized that eliminating this source of variance (year of maximum smoking) may affect heritability, however heritability estimates were unchanged and several of the same parametric and VC LOD score peaks were seen.

To our knowledge, no previous studies of any component of smoking behavior have indicated linkage with markers on chromosome 20 [1-3], with the exception of other analyses of these data [reviewed in [14]]. Our peak parametric LOD score of 1.98 at 70.32 cM on chromosome 2 is relatively near a chromosome 2p12 peak (89.2 cM) seen in a previous genome scan of ever/never smoking [2]. Our chromosome 17 peaks (parametric LOD at 66.85 cM and VC LOD at 138.03) are not near linkage peaks of other studies and are likely too far away to represent linkage to the candidate serotonin transporter gene (36.4 cM). In contrast to previous reports, we did not find support for linkage on chromosome 5 [1,2].

In conclusion, these results, though not definitive, may lend support to other evidence for a genetic influence on smoking behavior and may provide some clues as to where specific QTLs might lie. Additional research is needed to further understand the complex relationship of genes, the environment, and smoking behavior. Given that the best analysis method for a complex trait is often unknown, it is of particular interest whether the use of multiple methods with a conclusion based on the consistency of the findings would improve the reliability of linkage analyses.

## Figures and Tables

**Table 1:** Two-point LOD scores for maximum number of cigarettes per day.^A^

			Parametric Analysis^D^			Variance Components Analysis		
			
Chr^B^	cM^C^	Marker	Un- adjusted (θ)	Sex, age adjusted (θ)	Sex, age, year adjusted (θ)	Un- adjusted	Sex, age adjusted	Sex, age, year adjusted
1	167.15	GATA12A07	0.54 (0.3)	0.21 (0.3)	0.37 (0.3)	**1.28**	0.89	0.44
1	187.86	GGAA5F09	0.57 (0.3)	0.21 (0.3)	0.75 (0.3)	**1.46**	**1.34**	0.82
1	193.02	GGAA22G10	0.90 (0.3)	0.44 (0.3)	0.57 (0.3)	**1.13**	0.94	0.54
2	70.32	ATA4F03	**1.98 (0.2)**	**1.61 (0.2)**	0.80 (0.3)	0.04	0.07	0.01
2	100.60	GATA69E12	0.75 (0.3)	0.68 (0.3)	0.69 (0.3)	0.55	**1.02**	0.99
2	237.47	GATA4G12	0.03 (0.4)	0.00	0.00	0.99	**1.09**	0.71
7	62.68	GATA31A10	0.08 (0.4)	0.00	0.00	**1.10**	0.80	0.64
8	24.63	GATA25C10	0.05 (0.4)	0.06 (0.4)	0.02 (0.4)	0.45	0.74	**1.04**
11	58.51	ATA1B07	0.84 (0.1)	0.74 (0.1)	0.60 (0.2)	0.81	**1.10**	0.97
12	53.27	ATA27A06	0.00	0.09 (0.4)	0.09 (0.4)	0.20	0.88	**1.12**
13	83.74	GATA43H03	0.00	0.01 (0.4)	0.58 (0.3)	0.54	0.64	**1.06**
15	65.52	GATA151F03	0.83 (0.3)	**1.09 (0.2)**	0.67 (0.3)	0.49	0.21	0.02
17	66.85	GATA25A04	**1.52 (0.2)**	**1.39 (0.2)**	0.77 (0.3)	0.81	0.69	0.19
17	138.03	217yd10	0.05 (0.4)	0.12 (0.4)	0.08 (0.4)	**1.12**	**1.35**	**1.08**
20	70.51	GATA47F05	0.82 (0.3)	**1.30 (0.2)**	**1.19 (0.3)**	0.36	**1.07**	**1.39**
20	84.62	321xd1	0.61 (0.2)	**1.02 (0.2)**	**1.24 (0.2)**	0.77	0.95	0.98

^A^Markers shown have a LOD = 1. Bold, LOD > 1. ^B^Chr, chromosome. ^C^cM, sex-averaged distance from p-term. ^D^Parametric LODs are maximized over θ.
